# The tectorial membrane has a critical role in metabolic age-related hearing loss

**DOI:** 10.1016/j.ebiom.2025.105976

**Published:** 2025-10-18

**Authors:** Sonal Prasad, Marja Pitkänen, Anders Fridberger

**Affiliations:** Department of Biomedical and Clinical Sciences, Linköping University, Linköping, Sweden

**Keywords:** Ageing, Hearing loss, Sensorineural, Calcium, Presbycusis, Tectorial membrane

## Abstract

**Background:**

Millions of older adults have age-related hearing loss (ARHL), a disorder where potassium-secreting cells in the cochlea's lateral wall often degenerate. The degeneration reduces the force that drives ions into the sensory cells during sound stimulation, which is traditionally thought to explain the loss of hearing. Here we describe previously unknown mechanisms underlying this metabolic form of ARHL.

**Methods:**

Fluorescence spectroscopy and live-cell imaging was used in Dunkin-Hartley guinea pigs of either sex to investigate the effects of lateral wall dysfunction on the hearing organ and its accessory structures. Critical findings were confirmed by studying samples of human temporal bones.

**Findings:**

Lateral wall dysfunction caused calcium levels in the inner ear to decline, a change that was most pronounced in the tectorial membrane, an accessory structure crucial for transmitting acoustic stimuli to sensory cell stereocilia. This calcium depletion deprived the sensory cells of an essential ion. Additionally, the tectorial membrane detached from stereocilia, significantly impairing their ability to respond to sound. Sound-evoked responses were further decreased by sustained contraction of the entire hearing organ.

**Interpretation:**

These findings establish the tectorial membrane as a key factor in metabolic ARHL, which needs to be considered when developing better diagnostic tools or treatments.

**Funding:**

10.13039/501100004359Swedish Research Council grants 2017-06092 and 2022-00548, Swedish Brain foundation grant FO2023-0171 and US National Institutes of Health grant R01DC000141-44.

**Research in context**

**Evidence before this study**

Age-related hearing loss is common among older adults and can result from several problems in the inner ear. The disorder is usually classified into three types:

• Neural: due to damage to auditory nerve fibres,
• Sensory: caused by the loss of the sensory cells that detect sound,
• Metabolic: involving degeneration of the cells in the cochlea's wall that help maintain the ear's internal environment.

In the metabolic type, often considered the most common one, the positive electrical potential normally found close to the sensory cells is much reduced—and without this positive potential, sensory cells cannot function normally. However, the possibility that degeneration in the cochlea's wall could affect the hearing organ in other ways has not been considered.

**Added value of this study**

We used a physiologically based animal model to investigate what happens when cells in the cochlea's wall stop working. Using advanced imaging techniques, we discovered that calcium levels near the sensory cells dropped. This is an important observation because calcium is a key regulator of sensory cell function.

We also found that the tectorial membrane—which helps transmit sound-evoked vibration to the sensory cells—often detached from the sensory cells. This detachment made it nearly impossible for sound to reach the sensory cells.

To confirm that these findings are relevant for the human disorder, we examined samples from people with metabolic age-related hearing loss. We saw the same tectorial membrane detachment, and the extent of detachment predicted the severity of hearing loss.

**Implications of all the available evidence**

By demonstrating a critical role for the tectorial membrane in metabolic age-related hearing loss, this study is a major step forward in our understanding of the disorder. The findings challenge the current classification of age-related hearing loss and suggest that the “metabolic” and “sensory” types may be more closely linked than previously thought. Understanding this new mechanism opens the door to better diagnostic tools and treatments that target not just the sensory cells, but also the structures that help sound reach them.

## Introduction

Ageing often comes with loss of hearing. This can isolate people from family and friends, leading to loneliness^1^ and an increased risk of depression,^2^ but age-related hearing loss (ARHL) is also a risk factor for cognitive decline,^3^ and some 20% of all dementia cases are thought to be related to ARHL.^4^ ARHL is a complex disorder where genes^5^, ^6^, ^7^ interact with environmental factors, such as loud sound exposure^8^^,^^9^ and ototoxic drugs.^10^ The basic mechanisms leading from these risk factors to the development of hearing loss later in life however remain unknown.^11^

A common form of ARHL, the metabolic type, is associated with degeneration in the cochlea's lateral wall,^12^^,^^13^ which houses the stria vascularis, a layered structure with basal, intermediate, and marginal cells. Marginal cells use KCNQ1/E1^14^ potassium channels and the sodium-potassium-chloride co-transporter^15^ (NKCC1, also known as SLC12A2) to continuously move potassium ions into the fluid that surrounds the hearing organ. To ensure the flow of potassium ions through the cell layers in the lateral wall, an intricate ensemble of gap junctions, transporters and ion channels is present.^16^, ^17^, ^18^, ^19^

When the lateral wall stops working, the reduced potassium secretion is thought to decrease the positive electrical potential normally found near the hair cells. This removes the “battery” that drives sensory transduction, leading to the hypothesis that “battery replacement” could be a way to restore hearing.^20^^,^^21^

The “battery hypothesis” however neglects other functional roles for the lateral wall, which abundantly expresses the calcium transporter PMCA1^22^ and the calcium-selective ion channels TRPV5 and V6,^23^^,^^24^ all of whom contribute to regulating inner ear calcium levels. Such regulation is functionally important: Decreasing calcium can sever the tip links,^25^ molecular assemblies of cadherin 23 and protocadherin 15^26^ that are essential for gating mechanically sensitive ion channels in hair cells, while partial channel block and changes in resting open probability^27^ follow when calcium increases.

Calcium levels in the inner ear change after loud sound exposure,^28^^,^^29^ which has a clear association with the development of ARHL.^8^ There is ubiquitous expression of calcium transporters in the lateral wall, and calcium is important for regulating sensory cell function, leading us to think that calcium changes could underlie metabolic ARHL. To evaluate this hypothesis, an animal model was used, and critical findings were confirmed by studying samples from the human inner ear.

We show that loss of lateral wall function decreases inner ear calcium levels, with the most pronounced change occurring in the tectorial membrane, an acellular structure in contact with stereocilia. The reduced calcium level depressed sensory cell function, which was further decreased by sustained contraction of the hearing organ and by detachment of the tectorial membrane from stereocilia. Because the tectorial membrane is critical for conveying acoustic stimuli to stereocilia, its detachment contributes very strongly to hearing loss. Tectorial membrane detachment was also found in patients with metabolic ARHL, where the detachment predicted the extent of hearing loss. These findings establish the tectorial membrane as a key player in ARHL pathogenesis.

## Methods

Metabolic ARHL is defined by degeneration in the cochlea's lateral wall. The effects of such degeneration can be mimicked by blocking the sodium-potassium-chloride co-transporter^30^^,^^31^ (NKCC1), which reduces potassium secretion while damaging the cells in the stria vascularis.^32^

**Fig. 1:**
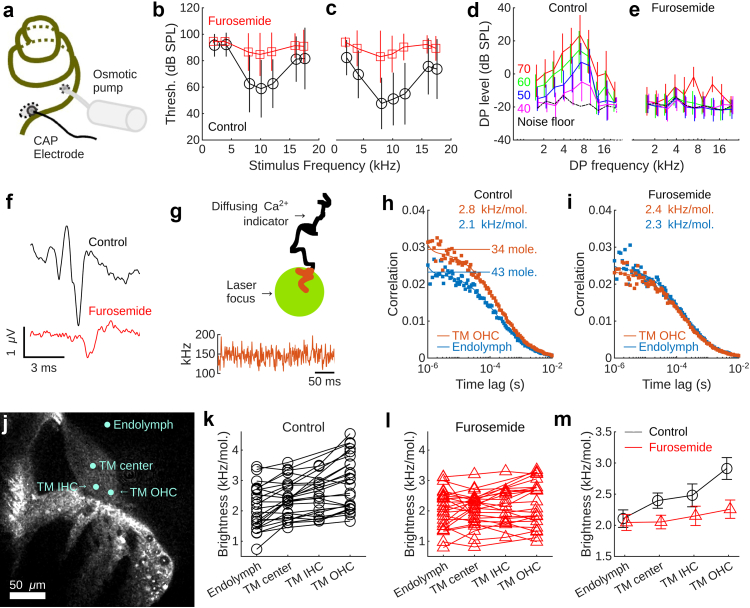
**Lateral wall dysfunction decreases tectorial membrane calcium. a**. An osmotic pump inserted in scala tympani of the basal turn of the cochlea delivered NKCC1 blockers or solvent. Compound action potential (CAP) audiograms were measured with a silver wire on the round window. **b and c**. The threshold (Thresh.) for the ABR (b) and CAP (c, 30 furosemide-treated animals and 15 controls) was elevated in animals given furosemide. dB SPL, decibels sound pressure level. Vertical bars denote the standard deviation. **d and e**. Distortion-product (DP) otoacoustic emissions (2F1–F2) in 15 controls (d) and 42 furosemide-treated animals (e). Vertical bars indicate the standard deviation. **f.** Animals in the furosemide groups that retained some auditory brainstem response had increased response latency. Stimulus level, 80 dB SPL. **g.** Principle of fluorescence correlation spectroscopy (FCS). Indicator molecules traverse the detection volume (green) through diffusion, are excited by a laser beam and start emitting light. From the fluorescence fluctuations that arise when molecules move, the number of molecules and their brightness can be determined by computing the autocorrelation of the fluorescence signal. **h.** Correlation curves from the tectorial membrane (TM) and the endolymph of a control animal. OHC, outer hair cell; kHz/mol, kilo-counts per second per molecule. **i.** Example correlation curves from the TM and endolymph in a furosemide-treated animal. **j.** Measurement positions. IHC, inner hair cells; OHC, outer hair cells. **k.** Controls had larger molecular brightness in the TM. mol. = molecule, other abbreviations as in panel j. **l.** Lack of brightness variation in the furosemide groups. Abbreviations as in panel k. **m.** Mean molecular brightness in the control and furosemide groups. Error bars show the standard error of the mean.

To study the effects of lateral wall dysfunction, we implanted guinea pigs—a species whose low-frequency hearing is similar to the human one—with osmotic pumps that delivered NKCC1 blockers to the cochlea for 7–10 days (Fig. 1a). Control animals received the buffered solution where the blocker was dissolved.

Results from the animal model were verified by studying human temporal bones and audiograms from the NIDCD National Temporal bone, Hearing and Balance Pathology Resource Registry.

### Ethics

The Regional Ethics Board in Linköping approved all animal procedures (permit 01122-2020). Animal care was under the supervision of the Unit for Laboratory Animal Science at Linköping University, ensuring compliance with Swedish regulations on the care and use of animals. Animals were housed in facilities approved by the Swedish Ministry of Agriculture. All animals had free access to food and water, cages featured an enriched environment, and animals were closely monitored after surgery. Animals that showed signs of distress according to a standardised assessment scale were euthanised. The reporting here conforms to the ARRIVE guidelines (https://arriveguidelines.org).

Procedures for removing human temporal bones were approved by the Massachusetts Eye and Ear Institutional Review Board (permit number 2019P003755). All participants provided written informed consent.

### Osmotic pump implantation

Young mature Dunkin-Hartley guinea pigs of either sex (250–300 g; 3–4 weeks old; Envigo, the Netherlands, catalogue number HsdDhl:DH) were used. To mimic lateral wall dysfunction, furosemide (Kronans Apotek, catalogue #130526) was delivered to the perilymph through an osmotic pump (pump rates were either 0.25 or 0.5 μl/h; Alzet catalogue #2004 or 2002). Ethacrynic acid (Merck Life Science, catalogue #SML1083) and bumetanide (Kronans Apotek catalogue #001009) were used in a separate series of experiments to ensure that the observed effects were not specific to the drug furosemide. The cannula of the pump was placed in scala tympani through a small cochleostomy in the basal turn. Each pump contained either 5 or 10 mg of furosemide per ml, leading to rates of drug delivery of either 1.25, 2.5 or 5 μg/h. In the results section, “low-dose” refers to animals given 1.25 or 2.5 μg/h; the “high-dose” group are those animals where the pump delivered 5 μg of furosemide per h. In the control group, osmotic pumps were filled with Ringer's acetate, the buffered solution used to dissolve furosemide. The infusion continued for 7–10 days. The dose levels are similar to the ones used in previous studies using this animal model (e.g. Schmiedt et al.,^31^ dose range 0.25–2.5 μg/h, 28 days).

To verify that furosemide caused hearing loss, auditory brainstem recordings were performed as described below. In addition, a silver wire electrode was placed in the round window niche before implanting the osmotic pump. This electrode was used for measuring the compound action potential (CAP) of the auditory nerve.

To reduce postoperative pain and discomfort, animals were treated with the non-steroidal anti-inflammatory drug carprofen (4 mg/kg body weight; Kronans Apotek #027647) beginning 30 min before surgery and continuing for 48 h. Anaesthesia was induced through subcutaneous injection of fentanyl (0.05 mg/kg; Kronans Apotek #545281), midazolam (2 mg/kg; Kronans Apotek #058078) and dexmedetomidine (0.1 mg/kg; Kronans Apotek #021331). Supplemental doses of anaesthesia were regularly administered to maintain the animal in an areflexic state. Surgery was performed under strictly aseptic conditions, with the animal placed on a thermostat-controlled heating pad to maintain body temperature at 37 ± 1 °C. Before skin incision, the long-acting local anaesthetic bupivacaine (Kronans Apotek catalogue #169912) was administered subcutaneously around the planned incision site. A retroauricular skin incision of about 2 mm length was then made and soft tissues retracted until the temporal bone was visible. The auditory bulla was opened until the round window niche and the base of the cochlea were clearly visible.

For cochlear infusion, a hole of ∼0.2 mm diameter was drilled in the basal turn of the cochlea slightly lateral to the round window, allowing access to scala tympani, and a fine-tipped vinyl cannula inserted. A small drop of silicone rubber placed 0.5 mm from the tip prevented the cannula from moving too far into scala tympani while helping to seal the infusion hole. A sub-cutaneous pocket was formed between the scapulae to accommodate the osmotic pump. A 4 cm long CAP electrode was placed in close contact with the round window niche. The CAP electrode and the cannula was kept in place temporarily by securing it to the temporal bone with Histoacryl (Swemed #2059016) followed with being secured permanently using dental cement (Agnthos AB, #7508). The osmotic pump was attached to the cannula, taking care to avoid air bubbles inside the cannula tubing followed with accommodating the pump inside the pocket. The bulla opening was closed using dental cement. The compound action potential electrode and cannula were secured with a suture knot in the muscle tissue beside the bulla opening, and the incision wound closed with interrupted sutures. The time from initial incision to wound closure was typically 1 h. Administration of an antidote mixture of Naloxone (Kronans Apotek #112695; 0.02 mg/kg), Flumazenil (Kronans Apotek #036259; 0.1 mg/kg), and Atipamezole (Kronans Apotek #569863; 1.0 mg/kg) was given 2–3 min after completing the surgery. Animals were treated post-operatively with the opioid analgesic buprenorphine (Kronans Apotek #085494; 0.05 mg/kg) and carprofen was administered for 2 days after the surgery. 3.0 ml of Ringer acetate was administered before and after surgery to compensate for any fluid loss. The body temperature, heart rate, oxygen level and respiration rate were monitored throughout the surgical procedure.

A total of 109 animals underwent surgery, but 43 out of these were excluded from analysis because blood clots were found in scala tympani, contamination of the solution in the osmotic pump was evident, bubbles were seen in the tubing of the osmotic pump, or because damage occurred during preparation of the temporal bone preparation. These exclusion criteria were formulated during the initial surgical training period, when it became clear that no useful data could be recovered from such animals.

### Recording of acoustically evoked potentials

After 7–10 days of infusion through the osmotic pump, animals were given ketamine (Kronans Apotek #570101; 40 mg/kg) and xylazine (Kronans Apotek #023572; 10 mg/kg), and otoacoustic emissions, auditory brainstem responses and compound action potential audiograms measured by placing the anaesthetised animal inside a sound-attenuated, electrically-shielded recording booth.

Auditory brainstem responses (ABR) and compound action potential (CAP) audiograms were recorded differentially using stainless-steel subcutaneous needle electrodes. When recording ABRs, the positive electrode was placed at the vertex, the reference in the neck, and the ground electrode in the hind limb. When recording CAPs, the positive electrode was at the round window membrane. Computer generated tone bursts with 10-ms duration and 1-ms rise and fall time were presented to the ear through a closed-field acoustic system (MF1 speakers, Tucker–Davis Technologies).

Input–output functions were generated by varying the intensity of the stimulus (85, 75, 65, 55, 45, 35, 25 dB SPL in steps at 7 different frequencies 2, 4, 8, 10, 12, 16, 18 kHz). The recorded potentials were band-pass filtered (passband 300–3000 Hz), amplified 10,000× and sampled with a 24-bit analogue-to-digital converter (USB-4431, National Instruments; sampling rate 100 kHz) controlled by custom Labview software. Responses to tone bursts were averaged 100–500 times and stored for subsequent offline analysis.

Distortion-product otoacoustic emissions were generated using two primary stimulus tones, f1 and f2, with a frequency ratio of 1.2. The two pure stimulus tones were applied through different earphones and tubing simultaneously to avoid nonlinear interactions on earphone membranes. The probe was placed inside the left ear canal of the anaesthetised guinea pigs.

### Temporal bone preparation

After the functional characterisation of the cochlea through *in vivo* recordings, animals were decapitated while still deeply anaesthetised, and the temporal bone isolated using previously described procedures (e.g. Fridberger et al.^33^). The isolated preparation retains the passive mechanics of the organ of Corti and generates cochlear microphonic potentials with amplitude and frequency tuning similar to those recorded *in vivo*.^34^ When the preparation is isolated from the animal, the endocochlear potential drops from its normal value of +60 mV. In the control group, the mean endocochlear potential, measured when placing the electrode in scala media, was 3.55 ± 9.2 mV in 27 control preparations; the corresponding values in 40 furosemide animals was −0.44 ± 10.4 mV (p = 0.11; t-test). If current is injected in scala media, the endocochlear potential can be restored toward its *in vivo* value, and in this situation, good preparations show hallmarks of cochlear amplification.^35^ However, current injection was not used in the present study, since our primary intention was to study changes in scala media calcium and organ of Corti physiology and morphology.

To maintain cellular viability, oxygenated tissue culture medium (Fisher Scientific, product #15218384) was continuously perfused. The perfusion system consisted of a thin silicon tube inserted into the scala tympani of the basal turn through the opening previously created for the osmotic pump cannula. The silicon tube was connected to a reservoir; the flow being driven by gravity. The perfusing solution exited through a small opening in scala vestibuli of the apical turn. This opening permitted the cells of the organ of Corti to be visualised with water immersion optics and confocal microscopy. The apical opening also permitted an electrode to be advanced through Reissner's membrane, to measure electrical potentials evoked by sound.

All *ex vivo* measurements were performed at room temperature (22–24 °C) using an upright laser scanning confocal microscope (Zeiss LSM 780 Axio Imager with ZEN black software) with a 40× water immersion objective (Nikon CFI APO NIR, numerical aperture 0.8).

### Electrophysiological recordings

A microelectrode was pulled from a glass capillary (outer diameter 1.5 mm, inner diameter 0.86 mm) and filled with artificial endolymph containing (in mM): 1.3 NaCl, 31 KHCO_3_, 128.3 KCl, and 0.023 CaCl_2_ (pH 7.4, osmolality ∼300 mOs/kg adjusted with sucrose; chemicals came from Sigma–Aldrich). The tip was bevelled at 20–30° to a final resistance of 5–8 MΩ. The electrode was attached to a micromanipulator and inserted through Reissner's membrane by using a computer-controlled stepper motor. For voltage recording, chloride silver wires were located both inside the microelectrode (active) and in the cell culture medium of the sample holder (ground), contiguous with scala vestibuli. The recorded signal was amplified 10×, low-pass filtered with cut-off at 5 kHz (Ix1 amplifier, Dagan) and digitised at 10 kHz with a 24-bit A/D board (USB-4431, National Instruments) using custom Labview software.

The sound stimulation consisted of a series of tone bursts ranging from 60 to 820 Hz at the level of 70 and 80 dB SPL. The tuning curve was obtained by determining the response amplitude at each frequency by Fourier analysis. The sound frequency that produced the maximal response was used for subsequent measurements of stereocilia motion.

Sound was delivered to the isolated preparation through a loudspeaker coupled to a tube that closely fitted the ear canal. Calibrations of sound pressure levels were performed by a microphone positioned at the end of the tube, at the location of the tympanic membrane.

### Reagents

The following stock solutions were prepared and further diluted in artificial endolymph to the desired concentration: Di-3-ANEPPDHQ (Potentiometric Probes), 4 mM in pure DMSO diluted 100 times for use; Calcein-AM, cell permeant dye (Thermo Fisher Scientific #C3100MP), 4 mM in pure DMSO diluted 100 times for use; Furosemide 10 mg/ml diluted to 5 mg/ml in Ringer acetate for use (pH 7.15); Bumetanide 0.5 mg/ml; Ethacrynic acid, 10 mg/ml diluted to 5 mg/ml in pure DMSO for use. Note that the effective concentration in the endolymph is lower than the concentration in the pump because of mixing with the fluid in scala tympani.

### Fluorescence correlation spectroscopy (FCS)

The fluorescent calcium indicator Calbryte 590 (potassium salt, AAT Bioquest #20707) was added to artificial endolymph solution at 50 nM concentration. The solution was introduced from the microelectrode to scala media with gentle pressure injections (∼10 psi, Picospritzer II, Parker Hannifin Inc) until the fluorescence count rate in scala media reached ∼100 kHz. The dye was left to diffuse for at least 3 min before recordings. Since FCS requires access with high-numerical aperture lenses, these measurements can only be performed at the apex of the cochlea.

Fluorescence signals were recorded at three positions within the tectorial membrane and one position in the endolymph in each preparation. At each location, 10 s recordings were repeated 7–8 times consecutively. Further data analysis was performed in Matlab (R2023a, the Mathworks, Natick, MA, USA). The autocorrelation of the signal was calculated,^36^ and the resulting correlation curves averaged over repetitions for each location. A three-dimensional anomalous diffusion model was fitted to the correlation curves, as described by Banks and Fradin^37^:

G(t)=1N∗1(1+(tτD)α)(1+(1S2)(tτD)α)12

where N is the average number of fluorescent molecules in the detection volume, t represents the time lag of the autocorrelation, $\tau_{D}$ is the average diffusion time through the detection volume, α quantifies the anomalous diffusion, and S is the aspect ratio of the detection volume. When appropriate, a term accounting for the effects of triplet state formation was also included. The molecular brightness of the indicator, which depends on the free calcium concentration, was determined by dividing the mean fluorescence count rate with the mean number of molecules (N), after correcting for the background fluorescence (which was determined before injecting the indicator into scala media).

### Confocal imaging

Confocal images were obtained in two different configurations. Reflection confocal imaging, a label-free technique suitable for living tissue, was used to visualise the tectorial membrane, which is not labelled by commonly used dyes. Fluorescence imaging with Calcein-AM and Di-3-ANEPPDHQ was used for visualisation of cellular structures, including hair cell stereocilia (dye concentrations of 2–4 μM were used). These dyes were added through the perfusion system after completing FCS measurements.

For most preparations, focus stacks were acquired at 12-bit pixel depth, 512 × 512 pixels, with an integration time of 6.30 μs per pixel, pinole of 1.0 Airy units and 1–3 μm section spacing for a total depth of 100 μm. Images were processed in ImageJ 2.9.0/1.53t software, ZEN 2012, and Matlab. Reflection images were acquired before the introduction of any dyes and fluorescence confocal imaging was performed after completing FCS measurements.

### Time-resolved rapid confocal imaging

To measure sound-evoked stereocilia motion, the hearing organ was stained with Di-3-ANEPPDHQ and Calcein-AM added through the perfusion system. Stereocilia were also stained with Di-3-ANEPPDHQ dissolved in the electrode solution. Preparations that showed dye diffusion into the scala vestibuli were discarded, since this can only occur if Reissner's membrane is broken. The preparation was stimulated acoustically near the bundles' best frequency (180–220 Hz) at 85 and 65 dB SPL. The frequency was selected from the largest peak of the tuning curve of the cochlear microphonic recordings. Custom Labview-based data acquisition software ensured that the exact phase of the acoustic stimulus with respect to each individual pixel in the confocal image is known, making it possible to reconstruct the motion of the sensory cells.^38^ The resulting image sequences were low-pass filtered and wavelet-based optical flow analysis was used to estimate motion for each pixel in the image sequence. To improve the signal-to-noise ratio, trajectories for all pixels in a 5 × 5 region were averaged. The pixel size was adjusted to allow measurement of motions down to ∼30 nm. When imaging bundle movements, each experiment began by acquiring a baseline of 2 sets of images over a period of 15 min.

### Human temporal bone morphology

Morphological analyses were performed on a collection of donated human temporal bones. This database does not include information about race or ethnicity, nor on other sociodemographic factors. In brief, each temporal bone was fixed by immersion in formalin for 1–2 weeks. Samples were then decalcified, dehydrated, and embedded in 12% celloidin, as described in detail by Schuknecht.^39^

Audiograms from each patient were algorithmically classified in one of three groups by Kaur et al.^40^; we adopted their classification, which was kindly shared by Dr. M. Charles Liberman. For the present study, we excluded participants with high percentage of surviving strial cells (i.e. the sensory ARHL group defined by Kaur et al.). This left 90 patients and 18 normal-hearing controls.

Morphological analyses were performed by an investigator blinded to the patient status. Using custom Matlab software, regions of interest corresponding to the tectorial membrane and organ of Corti were outlined on each image; the position of the basilar membrane and Reissner's membrane was marked by tracing them with lines. Since the animal experiments evaluated the apex of the guinea pig cochlea, we restricted morphometric analyses to the two most apical organ of Corti locations visible in the mid-modiolar sections we analysed. These measurements revealed two patients with no identifiable tectorial membrane in either of the two apical locations; another was excluded because of complete organ of Corti degeneration at the apex. Hence, 87 patients and 18 controls were included in the final analysis. Since we included all cases in the database that fulfilled the criteria listed above, power calculations were not performed.

In subsequent analyses, an automated algorithm determined the shortest Euclidean distance between the tectorial membrane and the surface of the hearing organ; areas of the organ of Corti and tectorial membrane were computed from the pixel size of each image and the number of pixels included in the corresponding region of interest. The two measurements from each cochlea were averaged, and the averaged value used for statistical analysis.

### Statistics

No prior animal studies examined calcium changes, stereocilia motion, or used live-cell imaging in animal models of age-related hearing loss. Hence, there was no data available to use for power calculations, and none were performed. However, the resource equation method^41^ gave E values ranging between 47 and 70, indicating that the study was powered to detect small differences. In the animal study, Drs. Prasad and Pitkänen were aware of the group assignments at all stages of the experiment (furosemide effects on morphology and thresholds could not be masked). Randomised group assignment was not used but animals came from the same breeder, they were of the same species, weight and age range, housed under identical conditions and receiving the same food, which minimises common sources of variability.

Human data were blinded during analyses. The sample size in the human part was outside of our control; all samples fulfilling the criteria listed in the preceding section were included, and a power calculation was not performed.

For FCS data, a generalised linear model was fitted using the gls function in the nlme package of R (version 4.3.1). The recording site and the furosemide dose were included as predictors; the molecular brightness of the calcium indicator was the response variable. Different locations recorded from the same preparation were expected to be correlated, which was considered by introducing a symmetrical correlation structure.

Apart from the generalised linear model, all other statistical tests were performed using Matlab's statistics toolbox (R2024b). Data were analysed using the anova function, followed by the Tukey–Kramer post-hoc test in those cases where significant differences were found. For data that were not normally distributed, the ranksum or Kruskal–Wallis tests were used.

Human data was analysed using the statistics toolbox in Matlab. Age, sex, tectorial membrane areas, organ of Corti areas and the gap between the organ of Corti and the tectorial membrane were analysed using one-way analysis of variance (anova); subsequent post-hoc comparisons were done with the Tukey–Kramer test.

A linear regression model was fitted to the human data using the ordinary least-squares approach, as implemented in the Matlab function fitlm. The dependent variable was the hearing threshold at 250 Hz. In the initial fit, predictor variables included age, sex, organ of Corti area, and the distance between the tectorial membrane and the organ of Corti. Since neither sex nor the organ of Corti area were significant predictors, the final model included only age and gap size as predictors.

### Role of funders

Funders were not involved in design, data collection, analyses, interpretation or writing.

## Results

### Lateral wall dysfunction decreases inner ear calcium levels

As expected, lateral wall dysfunction caused hearing loss. Fig. 1b shows elevated auditory brainstem response thresholds in animals that received the NKCC1 blocker furosemide (red, n = 39) compared to controls (black, n = 21); a similar pattern was evident in compound action potential audiograms (Fig. 1c). Distortion-product otoacoustic emissions, which gauge the function of one class of sensory cell, the outer hair cell, were also reduced (Fig. 1d and e). In cases where some auditory brainstem responses were present, the response was delayed compared to the control group (Fig. 1f), confirming a substantial hearing impairment (physiological measurements performed in the non-operated ear as well as additional measurements are shown in Supplemental Fig. S1).

Having confirmed that lateral wall dysfunction causes hearing loss, we next used FCS (Fig. 1g) to determine whether inner ear calcium levels were altered. Correlation curves recorded from control animals (Fig. 1h) had similar shape as those recorded from animals that received furosemide (Fig. 1i), but calcium levels of the furosemide-treated animals were lower, with the most pronounced change occurring in the tectorial membrane, an accessory structure critical for transmitting acoustic stimuli to the sensory cells.^42^

To systematically assess differences, calcium levels were measured at three positions in the tectorial membrane and a single position in the endolymph (Fig. 1j). In control animals, the tectorial membrane had higher calcium than the endolymph (Fig. 1k; n = 25), but animals with lateral wall dysfunction showed a different pattern (Fig. 1l, n = 24), with calcium reduced to endolymph-like levels at all locations (Fig. 1m). The difference between groups was significant (generalised linear model, p = 0.03; each unit increase of the furosemide concentration was associated with a change in the count rate of −61 photons per second per molecule, with the 95% confidence interval (CI) ranging from −115 to −6). Hence, loss of lateral wall function worsens hearing thresholds (Fig. 1b–f) and decreases calcium (Fig. 1m). In addition, when acquiring FCS data, it became evident that the morphology of the hearing organ was altered in animals that received furosemide.

### Lateral wall dysfunction contracts the organ of Corti and detaches the tectorial membrane from stereocilia

**Fig. 2:**
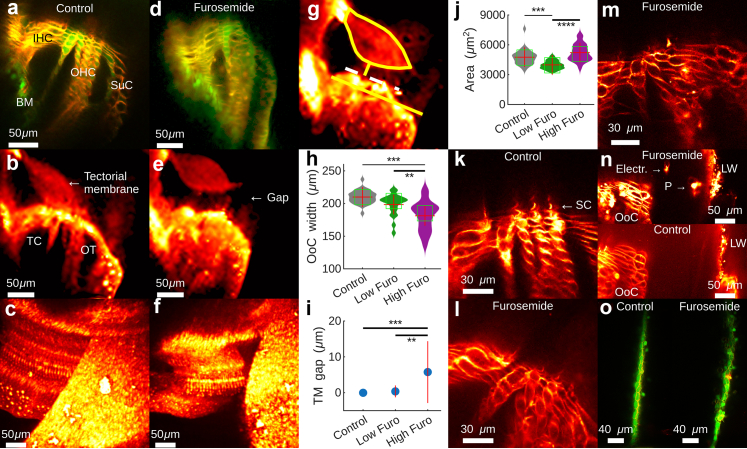
**Lateral wall dysfunction produces organ of Corti contraction and tectorial membrane detachment. a–f**. Fluorescence (a and d), reflection (b and e), and maximum brightness projections (c and f) show normal structure in controls and altered structure in the furosemide groups. IHC, inner hair cells; OHC, outer hair cells; BM, basilar membrane; SuC, supporting cells; TC; tunnel of Corti; OT, outer tunnel. **g**. Morphological changes were quantified by drawing a region of interest over the tectorial membrane (solid yellow region); a line drawn parallel to the surface of the hearing organ (the reticular lamina, white dashed line) was used to measure the distance between the tectorial membrane and the reticular lamina. The organ of Corti width is marked by the long yellow line. **h**. Organ of Corti (OoC) width in control animals (left), in animals receiving low and high furosemide (furo) dose. Red crosses denote the mean OoC width. The median is at the centre of the green box. ∗∗ indicates p = 0.005; ∗∗∗ indicates p = 2 × 10^−7^. **i**. Gap between the organ of Corti and the tectorial membrane (TM). Blue circles show the mean, red lines standard deviation. ∗∗ indicates p = 0.002; ∗∗∗ indicates p = 0.0002. **j**. TM area changes. ∗∗∗ indicates p = 0.0015; ∗∗∗∗ indicates p = 2 × 10^−6^ (30 controls, 22 high-dose, 19 low-dose). **k**. Bright stereocilia (SC) labelling in control animals; less intense labelling in animals with lateral wall dysfunction (**l**). **m**. Giant stereocilium in a furosemide-treated animal. **n**. Top image shows strial pathology in a furosemide-treated ear. Bottom image shows lack of observable pathology in a control ear. OoC, organ of Corti; Electr., electrode used for recording cochlear microphonic potentials; LW, later wall; P, cellular protrusion. **o**. Cells in Reissner's membrane were swollen and the structure appeared less regular in animals with lateral wall dysfunction. Green colour is the live/dead stain Calcein-AM; red colour is from di-3-ANEPPDHQ.

The isolated preparation permits detailed morphological studies on the living cells of the hearing organ. Dual staining with the live-cell marker Calcein-AM (green in Fig. 2a) and the membrane dye (Di-3-ANEPPDHQ, red in Fig. 2a), combined with reflection confocal imaging, revealed that the tectorial membrane was attached to the surface of the organ of Corti in control animals, the fluid spaces within the organ of Corti were intact (Fig. 2b), and three-dimensional reconstructions showed normal structure (Fig. 2c).

Starkly different images were obtained from animals where lateral wall function was inhibited. The fluid spaces within the organ of Corti were reduced (Fig. 2d), and a gap often appeared between the tectorial membrane and the surface of the hearing organ (Fig. 2e). Three-dimensional reconstructions showed contracted hearing organs (*cf*. Fig. 2f and c; similar results were achieved regardless of the NKCC1 blocker used, Supplemental Fig. S2a–c).

To quantify these changes, a region of interest was drawn over the tectorial membrane (Fig. 2g) and the surface the hearing organ outlined (white dashed line in Fig. 2g). The smallest distance between the structures was then measured (short yellow line in Fig. 2g). The long yellow line in Fig. 2g exemplifies how width was measured.

These measures revealed decreased organ of Corti width in animals receiving a high furosemide dose (5 μg/h; Fig. 2h; n = 22; Supplemental Fig. S2c) as compared to controls (n = 30; p = 2 × 10^−7^, analysis of variance followed by the Tukey–Kramer post-hoc test; F = 18.46; 70 df; mean difference 29 μm, CI 17–40 μm); there was also a significant difference in width between low (n = 19; 1.25 or 2.5 μg/h) and high furosemide doses (p = 0.005, Tukey–Kramer post-hoc test, mean difference 17 μm, CI 5–29 μm).

The distance between the reticular lamina and the underside of the tectorial membrane was larger in the high-dose group compared to the control group, where no animal had a measurable gap (Fig. 2i; p = 0.00014, analysis of variance followed by Tukey–Kramer post-hoc test, F = 10.1; 70 df; mean difference 6 μm, CI 2–9 μm); there was also a significant difference between high and low doses of furosemide (p = 0.002, anova followed by Tukey–Kramer post-hoc test; mean difference 5 μm, CI 2–9 μm). In the low-dose furosemide group, 1 of 19 animals (5%) showed tectorial membrane detachment; this feature was evident in 8 of 22 animals in the high-dose group (36%) but absent from all 30 controls.

Previous data^43^ prompted us to examine the area of the tectorial membrane. Analysis of variance showed a significant area difference between the groups (Fig. 2j; p = 4 × 10^−6^; F = 15; 70 df) but the details were complicated. The area was smaller in the low-dose group (p = 0.0015 by the Tukey–Kramer post hoc test; mean difference 780 μm^2^, CI 270–1300 μm^2^) whereas the high-dose group showed swelling compared to the low-dose group (p = 2.3 × 10^−6^; mean difference 1250 μm^2^, CI 700–1800 μm^2^) but no difference compared to controls (p = 0.068).

Other morphological changes were also apparent. In controls, stereocilia were brightly labelled (Fig. 2k), but labelling was often less intense in animals receiving furosemide (Fig. 2l). In some preparations, stereocilia with abnormal length and width were seen, suggesting fusion of hair bundles (Fig. 2m).

The lateral wall was difficult to visualise because of its proximity to the optically dense bone of the cochlear capsule, which tended to obscure the view. However, the top image in Fig. 2n is from an animal that received low-dose furosemide. The stria vascularis had an irregular outline with cells protruding into scala media (P in Fig. 2n). Such pathology was not observed in control animals (bottom image in Fig. 2n).

Cells in Reissner's membrane often looked swollen (Fig. 2o). This however did not compromise the membrane's barrier function, since most preparations exposed to furosemide produced cochlear microphonic potentials in response to acoustic stimulation.

We conclude that lateral wall dysfunction increases the risk for detachment of the tectorial membrane from stereocilia and leads to contraction of the organ of Corti. Next, we proceeded by examining the effects of such changes on sound-evoked electrical potentials and on the motion of stereocilia.

### Contraction makes the hearing organ less effective at deflecting stereocilia

**Fig. 3:**
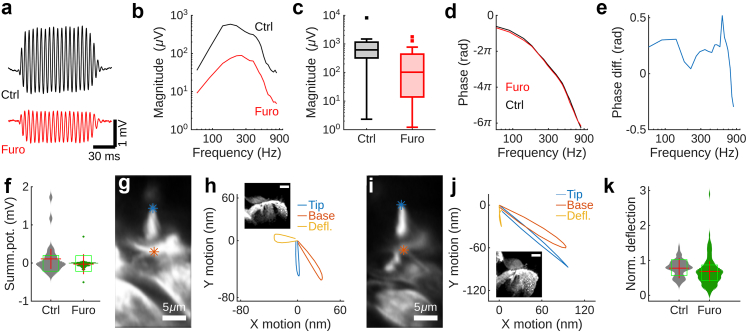
**Decreased cochlear potentials and stereocilia deflections. a.** Example waveforms recorded in response to sound at 200 Hz and 80 dB SPL. Furo, furosemide; ctrl, control. **b.** Median amplitude of the cochlear microphonic potential in 26 controls and 32 furosemide ears (these numbers apply to panels b–f). **c.** Peak values of the cochlear microphonic potential. **d.** Phases of the cochlear microphonic potential relative to the voltage driving the loudspeaker. Note the slight phase lag in the furosemide group. **e.** Mean phase difference (diff.) between controls and furosemide-treated ears. **f.** Amplitudes of the summating potential (Summ. pot.) at the peak of the cochlear microphonic potential. Red crosses denote the mean, the centre of green boxes show the median value. **g.** Outer hair cell stereocilia imaged during sound stimulation at 200 Hz and 80 dB SPL. Asterisks correspond to motion trajectories in panel h. **h.** Motion at the tip of the stereocilia bundle (blue) differs from the one at the base (red), producing deflection (yellow). Inset, reflection confocal image at the measurement site. Note normal appearance of fluid spaces inside the organ of Corti. Scale bar, 50 μm. **i.** Stereocilia from a furosemide-treated animal imaged during sound stimulation at 200 Hz and 80 dB SPL. Asterisks correspond to motion trajectories in panel j. **j.** Motion at the tip of the stereocilia bundle (blue) was nearly the same as the one at the base (red), resulting in small stereocilia deflection (yellow). Inset, reflection confocal image at the measurement site. Note contracted appearance of the organ of Corti. Scale bar, 50 μm. **k.** Normalised stereocilia deflection amplitude in controls and furosemide-treated animals. Red crosses denote the mean; the median is at the centre of the green square.

When stimulated by sound, sensory cells produce electrical potentials with a rapidly varying component at the stimulus frequency (the cochlear microphonic potential), superimposed on a sustained baseline deviation (the summating potential; Fig. 3a shows example waveforms).

The median amplitude of the cochlear microphonic potential was reduced across stimulus frequencies in animals with lateral wall dysfunction (Fig. 3b shows data with furosemide; Supplemental Fig. S2d and j show data with other NKCC1 blockers). The difference with the control group was statistically significant (Fig. 3c; p = 0.0008; ranksum test; Z value 3.37, ranksum 983). To examine whether the timing of the response to sound had shifted, we plotted the phase of the cochlear microphonic potential with respect to the voltage driving the loudspeaker (Fig. 3d; preparations with peak response below 10 μV were excluded). This revealed a slight phase lead in the control group (Fig. 3d) that was present at frequencies up to 700 Hz (Fig. 3e). The summating potential can be positive or negative depending on stimulus level and frequency,^44^ as seen in the distributions in Fig. 3f. We therefore compared absolute values; a significant reduction was found in animals with lateral wall dysfunction (Fig. 3f; p = 0.005 by the ranksum test, Z value 2.79, ranksum 946).

The decreased electrical potentials have severe consequences, since they reflect the sensory cell receptor potentials that drive cochlear amplification, a process that considerably improves hearing thresholds.

In principle, decreased sound-evoked electrical potentials could be caused by reduced stereocilia motion, by alterations in ion concentrations or channel conductance, or some combination of these variables. We therefore measured sound-evoked stereocilia motion using rapid confocal imaging.^33^ Stereocilia were labelled by injecting the dye Di-3-ANEPPDHQ through a microelectrode after replacing the electrode used during FCS measurements.

Fig. 3g shows an image acquired from a control animal during sound stimulation at 200 Hz and 80 dB SPL. The red asterisk marks the base of the bundle of stereocilia; the tip is marked with the blue asterisk. During sound stimulation, the bundle base moved along an inclined path (Fig. 3h, red trajectory), reaching a peak position 65 nm from the origin, whereas the bundle tip moved along a vertical path (blue trajectory). The differences between motion trajectories at the base and tip produces bundle deflection (yellow trajectory, 33 nm amplitude), which stimulates mechanically sensitive ion channels. This is the normal pattern of motion described in previous studies (e.g. Prasad et al.^45^). The inset in Fig. 3h shows a reflection confocal image from the same preparation, where fluid spaces inside the organ of Corti are visible and the tectorial membrane is in its expected position.

Stereocilia from animals with lateral wall dysfunction showed abnormal motion. The tip of the bundle (blue trajectory, Fig. 3j) moved along a path similar to the base (red trajectory) and as a result, stereocilia deflection was sharply reduced (yellow trajectory in Fig. 3j; Supplemental Fig. S2e–i shows similar effects with other blockers); the vertically oriented bundle “deflection” that remains is unlikely to effectively stimulate mechanically sensitive ion channels. This happened despite increased overall motion amplitude: The maximal displacement at the tip of the stereocilia bundle in Fig. 3j was 110 nm and the base displacement 130 nm (for comparison, the displacement at the bundle base was 65 nm in Fig. 3h).

The reflection image in the inset of Fig. 3j suggests an explanation for the altered sound-evoked motion: While the slightly swollen tectorial membrane retained its normal position, the hearing organ had contracted to the point where the internal fluid spaces were nearly obliterated.

Overall, normalised stereocilia deflections were significantly smaller in animals with lateral wall dysfunction (Fig. 3k; p = 0.002; Kruskal–Wallis test; 174 degrees of freedom, Chi-square 9.64; 12 control preparations and 22 furosemide-treated ears). Small stereocilia deflections are expected if the tectorial membrane has detached, as in Fig. 2e and i, but the data in Fig. 3i and j suggests that hearing organ contraction has a similar effect: It makes the organ of Corti ineffective, so despite increased overall motion, stereocilia deflection is reduced.

### Decreasing calcium makes the hearing organ less effective in converting sound to electrical potentials

**Fig. 4:**
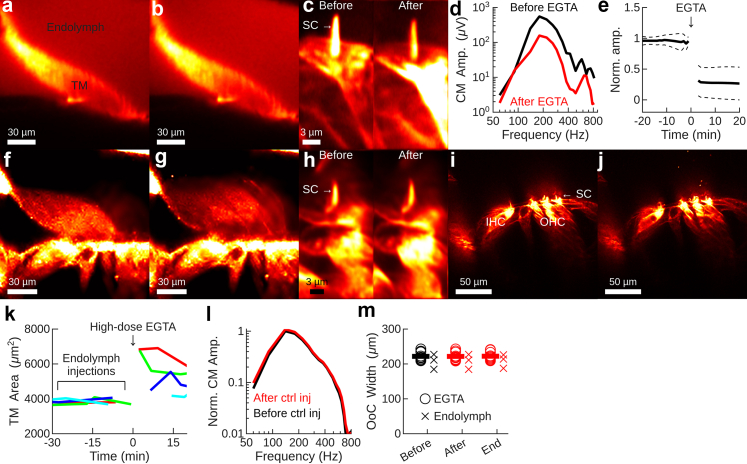
**Decreasing calcium reduces sound-evoked responses. a and b.** Tectorial membrane (TM) morphology before (a) and after (b) injection of a small amount of EGTA in the endolymphatic space. **c.** Small EGTA injections did not alter stereocilia (SC) morphology, although a minor decrease in staining intensity occurred. **d.** Tuning curves for the cochlear microphonic potential (CM) showed decreased amplitudes following small EGTA injections. **e**. The normalised peak amplitude of the cochlear microphonic potential decreased after low-dose EGTA. Solid black line indicates the means, dashed lines ±1 standard deviation. Data in panels a–e are replotted from Strimbu et al.^29^**f and g**. TM morphology before (f) and after (g) injection of a larger dose of EGTA. Note swelling in panel g. **h.** Stereocilia (SC) morphology before (left) and after (right) high-dose EGTA. **i and j.** Organ of Corti morphology is unchanged following EGTA (left image acquired before EGTA; right image afterwards). IHC, inner hair cells; OHC, outer hair cells. **k.** TM area in the 4 preparations where high-dose EGTA caused swelling. In two of these preparations, EGTA was preceded by multiple injections of endolymph. **l**. Endolymph injections did not change the cochlear microphonic potential (CM). **m.** Neither EGTA nor endolymph injections changed the width of the organ of Corti.

What are the functional effects of the reduced calcium shown in Fig. 1? This question already has a partial answer, for in a previous study^29^ we examined the effects of small endolymphatic injections of a calcium chelator (100 μM EGTA in artificial endolymph). Fig. 4a–d shows results from the previous study, demonstrating that these injections affected neither the tectorial membrane's area (*cf.* Fig. 4a and b) nor the morphology of stereocilia (Fig. 4c; apart from decreased intensity of staining, bundles appeared normal after EGTA). However, the cochlear microphonic potential was reduced at frequencies >100 Hz (Fig. 4d). Tracking the peak amplitude over time revealed an immediate decrease following EGTA injection (Fig. 4e; n = 10). Such decreases were not seen after control injections with endolymph alone.

To evaluate whether a higher EGTA dose produces morphological changes, new experiments were performed with 1 mM EGTA. At this dose, tectorial membrane swelling was observed in 4 out of 9 preparations (*cf*. Fig. 4f and g), but stereocilia appeared normal, with no observable morphological effect from the high-dose EGTA (Fig. 4h). The morphology of the organ of Corti also appeared normal after high-dose EGTA (*cf.* Fig. 4i and j).

Fig. 4k plots the tectorial membrane's area over time in the four preparations where swelling was observed. In two of these, EGTA was preceded by multiple injections of endolymph. Endolymph alone had no effect on the tectorial membrane's area or on cochlear microphonic potential amplitudes (Fig. 4l), and neither endolymph nor EGTA injections changed the width of the organ of Corti (Fig. 4m).

In summary, acutely decreasing endolymphatic calcium did not cause organ of Corti contraction or changes in stereocilia morphology but there was swelling of the tectorial membrane in some cases and, most importantly—a substantial decline in stimulus-evoked electrical potentials.

### Tectorial membrane detachment in human ARHL

**Fig. 5:**
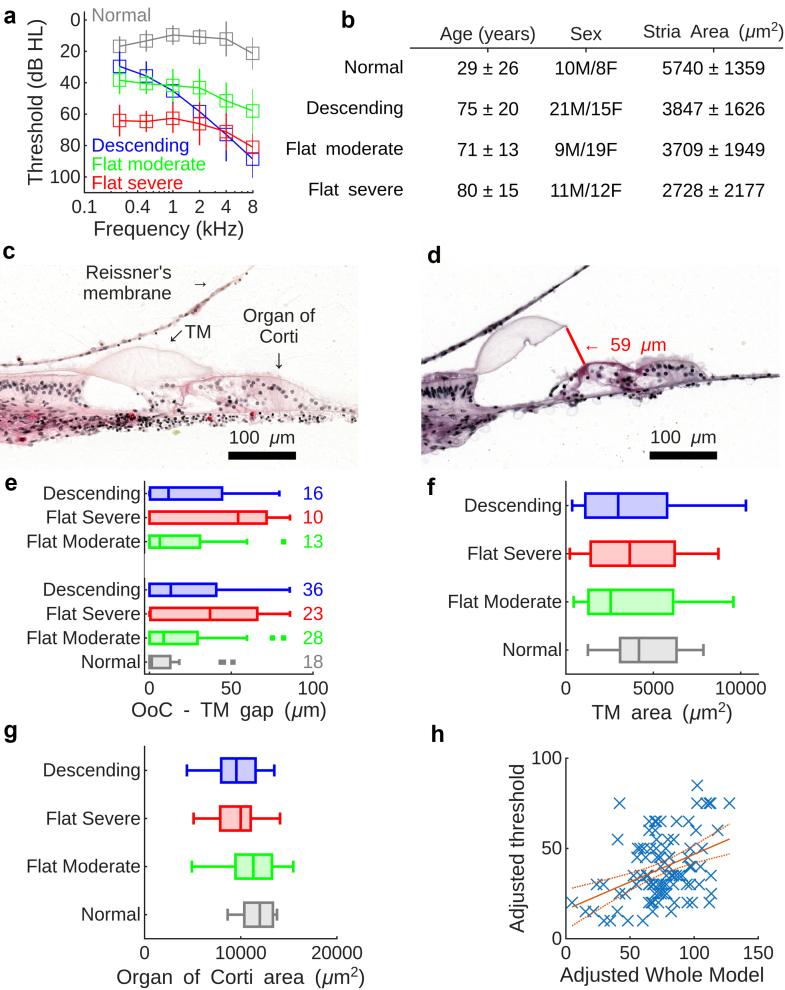
**Tectorial membrane detachment predicts human hearing thresholds. a.** Average hearing thresholds in normal-hearing participants and those with metabolic age-related hearing loss. **b.** Patient characteristics. Strial areas are mean values of the three most apical locations measured by Kaur et al.^40^ Values are means ± standard deviation. M, male sex; F, female sex. **c.** Representative image from the apex of the cochlea in a normal-hearing participant. TM, tectorial membrane. **d.** Image from a patient with hearing loss. The red line shows the distance between the tectorial membrane and the surface of the organ of Corti. **e.** Gap between the TM and organ of Corti (OoC). Numbers on the right is the number of patients. The bottom set of box plots show the entire dataset; top row shows the data after removing patients that had an identified cause that contributed to their hearing loss (such as loud sound exposure). **f.** TM areas in the four groups. **g.** OoC areas. **h.** Linear regression diagnostic plot, excluding the intercept term, showing how the adjusted hearing threshold at 250 Hz relates to the two adjusted predictors included in the final model. Red solid line is the linear regression, dashed lines show 95% confidence intervals. Statistical significance is indicated by the fact that the confidence intervals do not enclose any possible line with zero slope.

Because of its embedding deep within the temporal bone, neither the morphology nor the function of the cells in the human hearing organ can be evaluated during life. However, morphology can be evaluated post-mortem, in archived temporal bones. We therefore compared 87 donated temporal bones with lateral wall degeneration to 18 normal-hearing controls. Audiograms from each patient were classified in four groups by Kaur et al.^40^; we adopted their classification (Fig. 5a).

Demographic information is tabulated in Fig. 5b. The normal-hearing control group was younger than the three case groups (p = 1.4 × 10^−14^; anova; F = 31.8; 104 df; the normal-hearing group was 29 ± 26 years old; descending group 75 ± 20; flat moderate group 71 ± 13; flat severe 80 ± 15) and overall, there was a slight but non-significant female preponderance (p = 0.19; anova; normal-hearing group 44% female; descending group 42% female; flat moderate group 68% female; flat severe 52% female).

Since the physiological data were acquired at the apex of the guinea pig cochlea, we analysed the apex of the human cochlea, a region essential for speech encoding. The area of the stria vascularis at the apex was significantly smaller in the three patient groups (ranging from 2728 ± 2177 μm^2^ in the flat severe group to 3847 ± 1626 in the descending group, versus 5740 ± 1359 μm^2^ in the control group; p = 8.5 × 10^−6^ as compared to the normal-hearing group; anova; F = 9.92; 105 df), suggesting that all three groups had impaired strial function. Area differences between the three patient groups were not significant.

An image from a normal-hearing person is shown in Fig. 5c. Due to postmortem changes, there is some shrinkage of the organ of Corti, but shrinkage appeared more pronounced in people with ARHL, whose tectorial membranes frequently were in an abnormal position (Fig. 5d).

Next, an investigator blinded to the patient status drew regions of interest corresponding to the tectorial membrane and the organ of Corti on each image. In the normal-hearing group, the distance between the tectorial membrane and the surface of the organ of Corti was 10 ± 18 μm, but larger values were found in people with lateral wall degeneration (19 ± 23; 36 ± 30; 23 ± 26 μm; p = 0.009; anova, F = 4.03; 104 degrees of freedom; 44% of people in the normal-hearing group had an attached tectorial membrane versus 26–29% of the patients; Fig. 5e, lower set of bar graphs). Post-hoc testing indicated a significant difference between the normal-hearing and flat severe group (p = 0.007; Tukey–Kramer test; mean difference 26 μm, CI 6–47 μm).

Some patients had other conditions that may contribute to hearing loss, such as exposure to loud sounds and ototoxic drugs. Removing these participants, a difference in the gap size nevertheless remained (normal-hearing group 10 ± 18 μm; flat moderate group 20 ± 26 μm; flat severe group 43 ± 35 μm; descending group 24 ± 27 μm; top set of bar graphs in Fig. 5e; p = 0.03; F = 3.29; 54 degrees of freedom; anova), with the Tukey–Kramer test again indicating a significant difference between the normal-hearing and flat severe group (p = 0.015; mean difference 33 μm, CI 5–61 μm).

Differences in tectorial membrane area were not significant (Fig. 5f; p = 0.65; anova; 103 df), but the surface area of the hearing organ was larger in the normal-hearing group (Fig. 5g; p = 0.006; F = 4.44; 103 df; anova; normal-hearing group 11,700 ± 1700 μm^2^; flat moderate group 11,000 ± 2900 μm^2^; flat severe group 9500 ± 2300 μm^2^; descending group 9600 ± 2400 μm^2^). Post-hoc testing indicated significant area differences between normal-hearing people and those with a descending (p = 0.02; mean difference 2100 μm^2^, CI 200–4000 μm^2^) or flat severe audiogram (p = 0.03; mean difference 2200 μm^2^, CI 200–4200 μm^2^). This suggests organ of Corti contraction is a feature of human metabolic ARHL.

For a more complete picture, we used a linear regression model with age, sex, organ of Corti area and the distance between the organ of Corti and tectorial membrane as predictors and the hearing threshold at 250 Hz as the dependent variable. Although there was significant variability across patients, the model predicted the hearing threshold (p = 0.001; 92 error degrees of freedom, r^2^ = 0.18) but neither the organ of Corti area nor participant sex were significant (p = 0.15 and 0.77, respectively). Omitting these two factors resulted in a p value of 0.0003 (Fig. 5h), with age and the distance between the organ of Corti and the tectorial membrane being significant predictors (p = 0.003 and 0.04).

These data establish tectorial membrane detachment as an important factor in human ARHL.

## Discussion

This study demonstrates unexpected effects of lateral wall dysfunction. In animals, the tectorial membrane often detached from stereocilia, a change that reduced the ability to respond to sound. Calcium levels decreased, which contributed to a further decrease in the amplitude of sound-evoked electrical potentials, and the organ of Corti contracted, leading to additional reduction of sound-evoked responses. The combination of changes will cause considerable hearing loss, even if the sensory cells are otherwise functional.

In the human inner ear, it is not possible to assess the functional variables measured in the animal experiments, but morphological analyses showed an increased risk for tectorial membrane detachment in people with metabolic ARHL as compared to normal-hearing controls.

Conceivably, tectorial membrane detachment would require disruption of one or several of the proteins tubby,^46^^,^^47^ stereocilin,^48^ TMEM145^49^ (preprint), and otogelin,^50^ which link stereocilia to the tectorial membrane, but further studies are needed to clarify the exact mechanism.

Calcium ions regulate the resting open probability of mechanoelectrical transduction channels in hair cells^51^ and severe decreases in calcium can cut the tip links on which sensory transduction relies.^25^ Furthermore, the tectorial membrane buffers calcium near stereocilia^29^ in a manner dependent on the glycoproteins α- and β-tectorin.^52^ Detachment of the tectorial membrane will therefore change calcium dynamics around stereocilia in addition to reducing their sound-evoked motion. Interestingly, variants in the *TECTA* and *OTOG* genes contribute to both late-onset hearing loss^53^ and to familial Meniere's disease.^54^^,^^55^

The hearing organ contraction (Fig. 2) had interesting consequences, because the overall sound-evoked motion of the organ of Corti increased, despite decreased stereocilia deflections. The “effectiveness” of the hearing organ was reduced, meaning that larger motion produces less stereocilia deflection. Previous studies demonstrated that contraction of the organ of Corti occurs after loud sound^28^; this was suggested to be a part of a local mechanism for regulating cochlear sensitivity.^56^^,^^57^ Subsequent work established that the contraction response may be driven by activation of TRPA1 receptors on supporting cells.^58^ A corollary of this may be the decreased organ of Corti area apparent in human ears with ARHL.

In mice, mutations that impair lateral wall function^59^^,^^60^ cause profound hearing loss along with loss of hair cells, suggesting a trophic relationship between the lateral wall and the hair cells. However, neither the tectorial membrane's position nor its calcium content was examined in these studies in mice. Stereocilia abnormalities similar to the ones shown in Fig. 2 were seen in aged mouse cochleae,^61^^,^^62^ and Bullen et al.^61^ also noted tectorial membrane thinning and detachment in two temporal bone specimens from people with ARHL.

Study limitations include the use of a pharmacological animal model where hearing loss develops over the course of days rather than years. However, the hearing loss that does develop is quantitively similar to the hearing loss seen in quiet-reared, aged animals,^31^ which also involves damage to the lateral wall.^32^ Furthermore, there is no way of knowing whether the samples in the human database are representative of the larger population of people with ARHL, since relatively few people donate their temporal bones for research. Some 40% of samples in the database came from people with exposure to loud sounds or ototoxic drugs, which may produce a different pattern of hearing organ pathology than ageing alone. Nevertheless, it is reassuring that important findings from the animal model were confirmed in the human data.

A common classification of ARHL assumes that hearing loss can be caused by loss of sensory cells (the “sensory” category) or by dysfunction of the lateral wall (the “metabolic” variety; e.g. Lewis et al.^7^; Kaur et al.^40^). Here we demonstrated that lateral wall dysfunction has direct structural and functional effects on the organ of Corti and the tectorial membrane, so the “sensory” and “metabolic” categories are not distinct. Furthermore, these findings establish the tectorial membrane as a key player in metabolic ARHL.

## Contributors

Conceptualisation: AF; Methodology: SP, MP, AF; Investigation: SP, MP, AF; Funding acquisition: AF; Supervision: AF, SP; Writing—original draft: AF; Writing—review & editing: SP, MP, AF. FCS and animal morphometry data analysis was done by M.P., all other animal data were analysed by S.P.; A.F. used Matlab scripts from S.P. and M.P. to verify results and to prepare final figures. A.F. analysed human data, with M.P. and S.P. having full access to results, and M.P verifying the calculations. All authors read and approved the final version of the manuscript.

## Data sharing statement

A materials transfer agreement limits sharing of human data, but these data can be accessed by filing a request with the NIDCD National Temporal bone, Hearing and Balance Pathology Resource Registry at the email address tmeesmb2@meei.harvard.edu. The data displayed in Fig. 1, Fig. 2, Fig. 3, Fig. 4 can be downloaded from Mendeley Data (https://doi.org/10.17632/tyt5hbtjm4.1) or by emailing anders.fridberger@liu.se.

## Declaration of interests

Authors declare that they have no competing interests.
