# Supplementary material for: The tectorial membrane has a critical role in metabolic age-related hearing loss
Source: eBioMedicine. 2025 Oct 18;121:105976. doi: 10.1016/j.ebiom.2025.105976 (PMC12569810; doi:10.1016/j.ebiom.2025.105976)
Supplement: Supplementary Text and Figures [file mmc1.docx]

Supplementary Text

Effects of furosemide on the non-operated ear

Furosemide was applied locally to the left ear through an implanted osmotic pump, but it is possible that drug reaching the circulation could have systemic effects, including hearing loss on the non-operated right ear. To examine this possibility, the same set of *in vivo* physiological measurements were performed on operated and non-operated ears alike. Auditory brainstem response thresholds on the non-operated ear were nearly identical in animals receiving furosemide and those that did not (Fig. S1a). The timing of the response also did not differ between the groups (Fig. S1b) and input-output curves were close to identical (Fig. S1c).

Measurements of distortion-product otoacoustic emissions on the non-operated right ear however showed larger amplitude across stimulus levels in control animals (Fig. S1d) as compared to animals that received furosemide (Fig. S1e and f), a difference that was statistically significant (p values ranging from 0.0005 to 0.01; t-test). This may indicate that furosemide has subtle effects on outer hair cell function in the contralateral ear, but these effects are not substantial enough to result in elevation of auditory brainstem response thresholds (Fig. S1a - c).

Three-tone stimuli to assess cochlear function

A previous study (*50*) suggested that inner hair cells in the high-frequency regions of the cochlea encode the envelope of acoustic stimuli, even when the envelope is presented at frequencies far below the best frequency of the recording location. To examine whether loss of marginal cell function affects this capacity for low-frequency envelope extraction, we used the electrode on the round window membrane to probe the response to the stimulus utilized in reference 1.

In brief, the stimulus consists of three high-frequency sine waves with 500-Hz frequency difference ($f_{2}= f_{1}+f_{e}; f_{3}= f_{1}+2f_{e}$, where $f_{e}$ denotes the frequency separation between the three tones). When all three frequency components have zero phase, the envelope shows large peaks alternating with smaller ones (Fig. S1g; envelope marked with black line; $f_{e}$ also determines the frequency of the large envelope peaks). However, if the phase of the center component ($f_{2}$) is shifted by 90 degrees, the large peaks disappear and a flatter envelope results (black line on the lower waveform in Fig. S1g). It should be emphasized that this substantial shift in envelope shape arises solely because of intereference effects between the three frequencies present in the tone complex; there is no change in the frequency or sound pressure level of either $f_{1}, f_{2} or f_{3}$. This ability to change the envelope while maintaining a constant amplitude spectrum is the chief advantage of this stimulus.

When the phase of $f_{2}$is systematically shifted over a 180-degree range, the amplitude of the large peaks change (Fig. S1h); large peaks are absent at center phase zero. If systematic stimulus phase changes are used in an animal with an electrode on the round window membrane, the recorded electrical potentials will show a peak at the envelope frequency. Findings by Nuttall *et al* suggested this peak may depend more on inner than outer hair cell activation.

In control animals (Fig. S1i, black lines), the ear that received vehicle alone showed a pattern of amplitude changes consistent with the prediction in Fig. S1h. Ears that received furosemide did not show such amplitude variations (Fig. S1i, red curve). Further study is required to determine the relative role of inner and outer hair cells for this response. The result is consistent with other measures showing a large hearing impairment in ears that were infused with furosemide, and the absence of such impairment in control ears infused with vehicle alone.

Effects of bumetanide and ethachrynic acid on cochlear structure and function

Conceivably, the effects of furosemide described in the main text could result from non-specific drug effects rather than blockade of the NKCC1 transporter in the marginal cells. To exclude this possibility, further experiments were performed, where bumetanide or ethacrynic acid replaced furosemide in the osmotic pump. These substances block the NKCC1 transporter but are chemically different from furosemide.

Bumetanide (Fig. S2a and b; 0.5 mg/ml) resulted in morphological changes similar to the ones observed after furosemide. The fluid spaces in the organ of Corti were reduced in size; measurements of the width of the organ of Corti indicated no difference from animals treated with furosemide (Fig. S2c). The amplitude of the cochlear microphonic potential was reduced as compared to furosemide (Fig. S2d, n=11), possibly indicating that bumetanide has more severe functional effects than furosemide.

To further assess functional changes, we measured the sound-evoked motion of outer hair cell stereocilia in animals that received bumetanide (Fig. S2e – f). Neither the normalized tip motion, base motion, or deflection of stereocilia (Fig. S2g) was different in animals that had received received bumetanide, as compared to those that received furosemide.

Measurements performed with osmotic pumps loaded with ethacrynic acid gave similar results, both as regards stereocilia movement (Fig. S2h -i) and cochlear microphonic potentials (Fig. S2j).

In conclusion, organ of Corti contraction, altered stereocilia movements, and reduction of cochlear microphonic potential amplitudes result from drugs that block the NKCC1 transporter. These effects appear largely independent of the drug that is used, showing that non-specific drug effects are not likely to explain the findings described in the main text.





Fig. S1. Physiological effects of furosemide on the non-operated ear. Auditory brainstem response thresholds (Thresh.) in non-operated ears show no difference between control animals (n=20) and those that received furosemide (n=46). B. Response latencies were similar on the non-operated ear in animals that received furosemide and those that received vehicle alone. The stimulus was a 10 kHz tone burst at 75 dB SPL. C. Over the range 25 to 85 dB SPL, the peak amplitude of the auditory brainstem response at 10 kHz was similar on the non-operated ears of animals that received furosemide (n=46) and those that received vehicle alone (n=20). D-E. The mean amplitude of the 2F1-F2 distortion product otoacoustic emission in control animals (n=39) was larger on the non-operated ear than the amplitude recorded in animals that received furosemide (n=48), a difference that was significant (p=0.0005, t-test). F. The decrease in emission amplitudes is also evident when examining the increase in distortion product amplitude at F1=7.2 kHz with increasing stimulus level. G. Schematic showing the different envelopes (black lines) that result when changing the phase of the center component in a complex of three tones with equal frequency spacing of 500 Hz. The center phase is given on the left of each plot, the envelope is indicated by black lines, and the red lines show the fine structure of the acoustic stimulus. H. The amplitude of the big envelope peaks change in a predictable manner when the center phase is systematically altered. I. Ears that received vehicle (n=23) showed the expected pattern of envelope amplitude changes when the center tone phase was altered, but this was not the case in ears that received furosemide (n=33; stimulus level 57 dB SPL, $\boldsymbol{f}_{\boldsymbol{1}}$ = 8 kHz, $\boldsymbol{f}_{\boldsymbol{e}}$= 500 Hz).

.


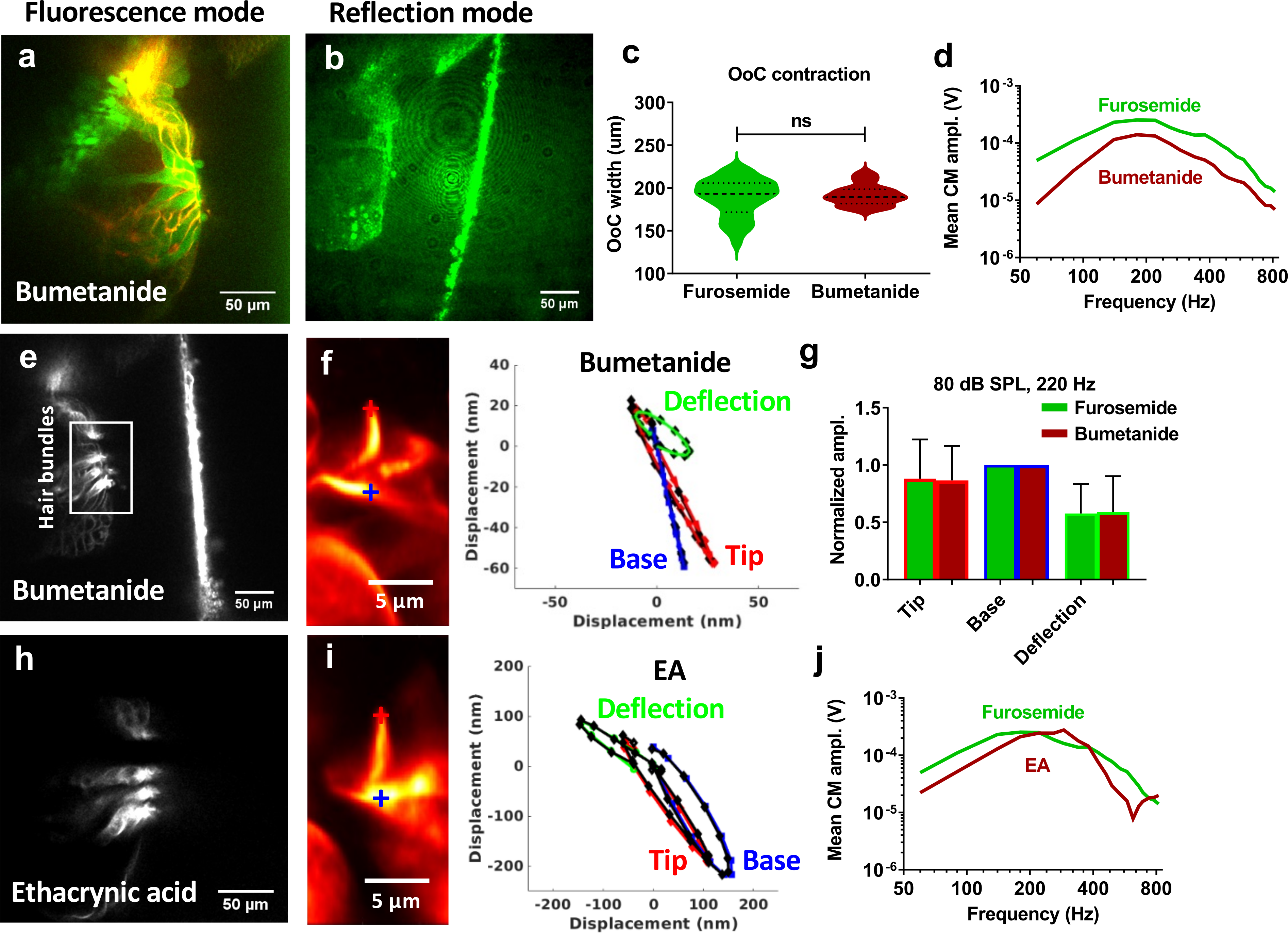


Fig. S2.

**Bumetanide and ethacrynic-acid-induced effects on cochlear potentials and stereocilia deflections.** (A, B) Confocal images of the organ of Corti acquired in fluorescence and reflection and mode showing a collapsed appearance of the organ of Corti similar to the one seen after furosemide. (C) The violin plot indicates similar reduction in organ of Corti (OoC) width in bumetanide- and furosemide-treated animals. (D) Tuning curves of the microphonic potential in 11 preparations shows reduced amplitude in animals treated with bumetanide (dark red line; 0.5 mg/ml concentration in the osmotic pump) as compared to furosemide (green). (E) Although the organ of Corti was contracted, stereocilia with normal morphology were present (F). The sound-evoked motion of the stereocilia tip (red) and its base (green) was similar to the one recorded after furosemide. Due to the similar tip and base motion, deflection of stereocilia was small (green). (G) The normalized average motion of outer hair cell stereocilia was similar in bumetanide- and furosemide-treated animals. Data were normalized to the base trajectory amplitude. Averaged data from 11 individual preparations. (H) Animals receiving ethacrynic acid showed hearing organ contraction, but stereocilia with normal morphology were present. (the concentration of ethacrynic acid in the osmotic pump was 5 mg/ml). (I) Sound-evoked motion of the stereocilia tip (red) and its base (blue) in an example preparation. Motion at the tip of the stereocilia bundle is nearly the same as the one at the base resulting in smaller deflection of the stereocilia (green), resembling the findings after furosemide and bumetanide. (J) Tuning curves cochlear microphonic potential in 4 preparations showed amplitude similar to the one in animals treated with furosemide (EA, ethacrynic acid, dark red line; furosemide, green). When recording stereocilia motion, the stimulus was at 220 Hz tone at 80 dB SPL in all cases.
